# Development of a simple and miniaturized sandwich-like fluorescence polarization assay for rapid screening of SARS-CoV-2 main protease inhibitors

**DOI:** 10.1186/s13578-021-00720-3

**Published:** 2021-12-05

**Authors:** Gangan Yan, Dongsheng Li, Yuan Lin, Zhenghao Fu, Haiyan Qi, Xiaoping Liu, Jing Zhang, Shuyi Si, Yunyu Chen

**Affiliations:** 1 Institute for Drug Screening and Evaluation, Wannan Medical College, 241002 Wuhu, China; 2 Institute of Medicinal Biotechnology, Chinese Academy of Medical Sciences and Peking Union Medical College, 100050 Beijing, China; 3 State Key Laboratory of Bioactive Substances and Function of Natural Medicine, Institute of Materia Medica, Chinese Academy of Medical Sciences and Peking Union Medical College, 100050 Beijing, China; 4 Anhui Provincial Engineering Laboratory for Screening and Reevaluation of Bioactive Compounds of Herbal Medicines in Southern Anhui, Wannan Medical College, 241002 Wuhu, China

**Keywords:** SARS-CoV-2, Main protease inhibitor, 3CL protease inhibitor, Fluorescence polarization, High-throughput screening, Dieckol

## Abstract

**Background:**

Severe acute respiratory syndrome coronavirus 2 (SARS-CoV-2) is highly transmissible and has caused a pandemic named coronavirus disease 2019 (COVID-19), which has quickly spread worldwide. Although several therapeutic agents have been evaluated or approved for the treatment of COVID-19 patients, efficacious antiviral agents are still lacking. An attractive therapeutic target for SARS-CoV-2 is the main protease (Mpro), as this highly conserved enzyme plays a key role in viral polyprotein processing and genomic RNA replication. Therefore, the identification of efficacious antiviral agents against SARS-CoV-2 Mpro using a rapid, miniaturized and economical high-throughput screening (HTS) assay is of the highest importance at the present.

**Results:**

In this study, we first combined the fluorescence polarization (FP) technique with biotin-avidin system (BAS) to develop a novel and step-by-step sandwich-like FP screening assay to quickly identify SARS-CoV-2 Mpro inhibitors from a natural product library. Using this screening assay, dieckol, a natural phlorotannin component extracted from a Chinese traditional medicine *Ecklonia cava*, was identified as a novel competitive inhibitor against SARS-CoV-2 Mpro in vitro with an IC_50_ value of 4.5 ± 0.4 µM. Additionally, dieckol exhibited a high affinity with SARS-CoV-2 Mpro using surface plasmon resonance (SPR) analysis and could bind to the catalytic sites of Mpro through hydrogen-bond interactions in the predicted docking model.

**Conclusions:**

This innovative sandwich-like FP screening assay enables the rapid discovery of antiviral agents targeting viral proteases, and dieckol will be an excellent lead compound for generating more potent and selective antiviral agents targeting SARS-CoV-2 Mpro.

## Introduction

In December 2019, novel coronavirus disease 2019 (COVID-19), caused by severe acute respiratory syndrome coronavirus 2 (SARS-CoV-2), quickly became a global ongoing pandemic. To date, COVID-19 has spread worldwide, causing 207,173,086 confirmed cases and over 4,361,996 deaths updated on August 17, 2021. Considering a serious threat, the World Health Organization (WHO) has declared COVID-19 to be a public health emergency of international concern. Remdesivir was recently approved by the Food and Drug Administration (FDA) for the treatment of hospitalized patients with COVID-19, but the trail from WHO showed that remdesivir has little benefit or no impact on survival for COVID-19 patients [1, 2]. Therefore, no efficacious antiviral agents are currently available to fight against COVID-19.

SARS-CoV-2 is a small, enveloped virus containing a single-stranded positive-sense RNA genome with a length of ∼ 30,000 nucleotides [3]. In viral fusion and cell entry processes, an essential step for its infection heavily depends on a high affinity between the receptor-binding domain (RBD) of spike glycoprotein and the host cell surface angiotensin-converting enzyme 2 (ACE2) [4, 5]. After infection initiates, viral genomic RNA adequately utilizes the host ribosome to translate two polyproteins (pp1a and pp1ab), which are efficiently cleaved by the main protease (Mpro) and papain-like protease (PLpro) to release 16 non-structural proteins (nsps) for viral genomic RNA replication. During this proteolytic processing, Mpro is responsible for 11 specific cleavage sites to generate 12 nsps [6].

Mpro, a chymotrypsin-like cysteine protease (33.8 kDa), is activated and matured in a homo-dimerization form [7]. Each monomer features a catalytic site containing a cysteine nucleophile. In particular, SARS-CoV-2 and SARS-CoV share 96% sequence identity in their Mpro sequences while their genomes only share approximately 82% identity. Mpro is a highly conserved and pivotal enzyme in SARS-CoV-2 life cycle, and mutations in Mpro are often rare. Importantly, closely related homologues are absent in human cells [6, 7]. Therefore, Mpro may be a promising antiviral target for the design of broad-spectrum antiviral agents [8]. Recently, several Mpro inhibitors have been identified via structure-based virtual screening assay, fluorescence resonance energy transfer (FRET) assay, colorimetric screening assay, cell-based luciferase complementation screening assay, green fluorescent protein (GFP) splitting screening assay, and phenotypic screening assay [9–15]. Among these reported screening approaches, virtual screening assay is a computational method for the discovery of potential Mpro inhibitors, but it remains challenging to accurately define hot spots in Mpro and efficiently remove the false positive hits [16]. The prevalent FRET and colorimetric screening assays can well mimic the in vivo proteolytic process, but these enzymatic kinetics assays are usually unstable and expensive because of the large amounts of substrates consumed in a high-throughput screening (HTS). Moreover, due to auto-fluorescence interferences, natural products may quench the emission fluorescence of a FRET fluorogenic substrate, and false positive hits are inevitably present [17]. The cell-based luciferase and GFP splitting assays are frequently associated with a high screening cost, tedious cell culture, long screening cycles and poor repeatability. A high-cost phenotypic screening assay must be operated in biosafety level-3 (BSL-3) laboratory, and the complexity of this approach is not readily compatible with HTS pipelines. In addition, the target validation of positive hits remains extremely challenging [7]. At present, COVID-19 is becoming a serious health care crisis globally because frequent mutations of SARS-CoV-2 increase the infectivity to human and reduce the effectiveness of approved vaccines [18–20]. Thus, there is an urgent need to develop a simple, miniaturized and cheap HTS assay for rapid screening of novel antiviral agents targeting SARS-CoV-2 Mpro [21].

Here, we first combined the fluorescence polarization (FP) technique with biotin-avidin system (BAS) to develop and optimize a novel sandwich-like FP screening assay for quick identification of SARS-CoV-2 Mpro inhibitors. This innovative and step-by-step sandwich-like FP screening assay can be easily popularized to facilitate rapid large-scale screening of antiviral agents targeting viral proteases.

## Results

### Preparation of the highly active SARS-CoV-2 main protease

**Fig. 1:**
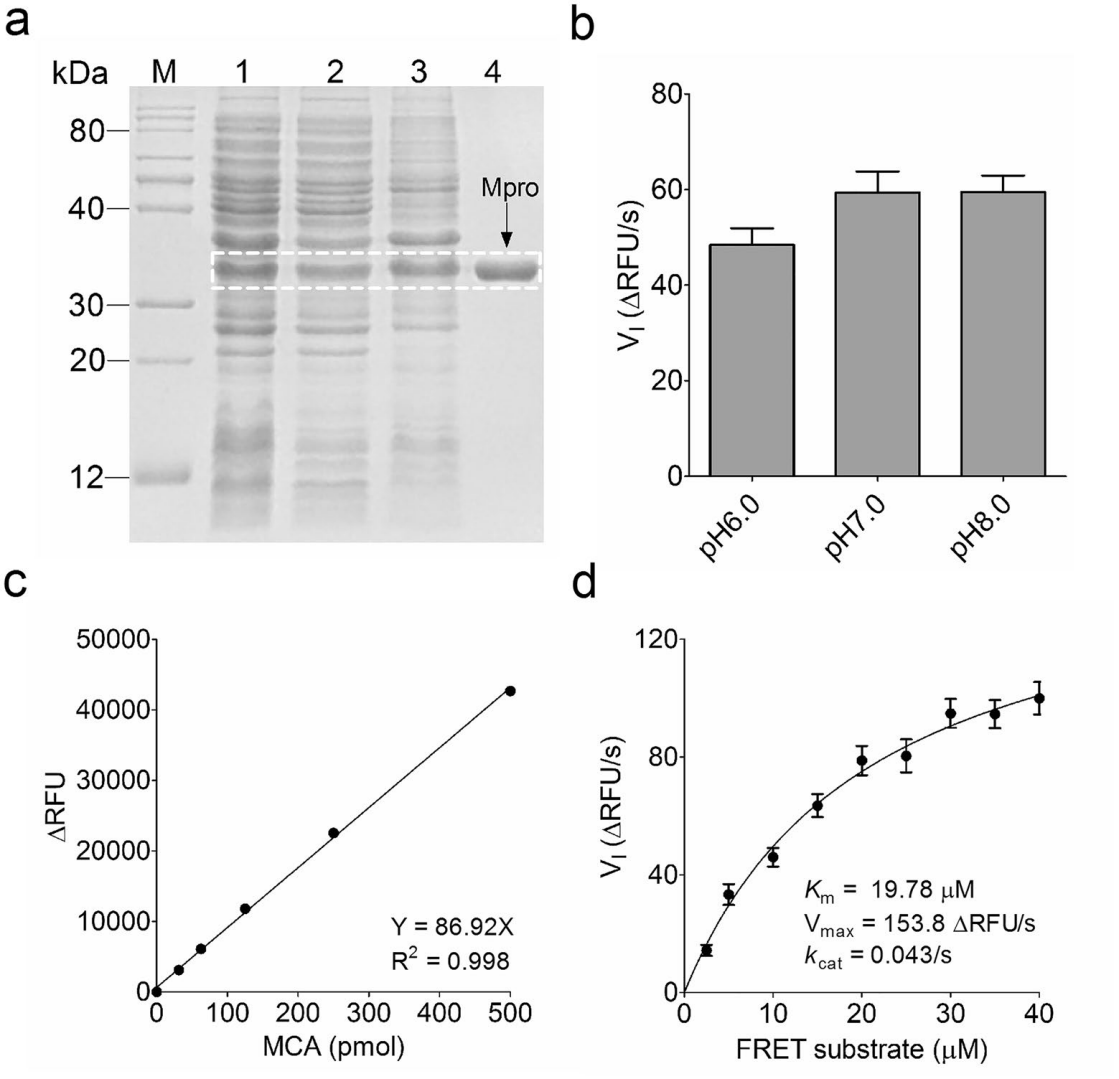
Preparation and characterization of SARS-CoV-2 main protease (Mpro). **a** Expression and purification of SARS-CoV-2 Mpro. The highly active Mpro was expressed in *E. coli* Rosetta (DE3) cells as the soluble protein after induction with 0.2 mM IPTG at 30 °C for 8 h. A HisTrap^TM^ chelating column was used to purify polyhistidine-tagged Mpro from *E. coli* cell extracts, and then the purity of purified Mpro was analyzed using SDS-PAGE. M: protein marker; 1: total cell extracts after IPTG induction; 2: supernatant of the cell lysate; 3: precipitated Mpro in 25% saturated ammonium sulfate solution; 4: purified Mpro band (34 kDa). **b** Reaction buffer optimization. The reaction mixture containing 0.25 µM Mpro and 10 µM FRET substrate was incubated in the indicated three buffers, and the change of RFU value was continuously recorded by a microplate reader (BioTek) using a FRET assay. The initial velocity (V_I_) of the proteolytic activity was calculated by a linear regression for the first 30 s of the kinetic progress, and the curve was plotted in GraphPad Prism 5.0. **c** Plotting the MCA standard curve to convert RFU value to the amount of the cleaved FRET substrate (pmol) using the derived equation. MCA: 7-methoxycoumarin-4-acetic acid. **d** Calculation of Mpro kinetic constant *K*_m_ and *k*_cat_ values in the FP assay buffer. The Michaelis-Menten equation of Mpro proteolytic kinetics was plotted using GraphPad Prism 5.0 according to the V_I_, and then *K*_m_, V_max_ and *k*_cat_ values were calculated

A codon-optimized SARS-CoV-2 Mpro gene was cloned into a pET-21a(+) vector, and the soluble Mpro was expressed in *E. coli* Rosetta (DE3) cells. Subsequently, the highly expressed Mpro was purified by a HisTrap^TM^ chelating column (Fig. 1a). In order to characterize its enzymatic activity and to determine a kinetic FRET assay condition, we designed a fluorescently labeled FRET substrate, MCA-AVLQSGFR-Lys(Dnp)-Lys-NH_2_, derived from the N-terminal auto-cleavage sequence of the viral protease [7]. We analyzed the Mpro proteolytic activity in 3 buffers with different pH and found that Mpro displays a highest velocity in pH8.0 buffer (Fig. 1b), which contains 10 mM Tris, 50 mM NaCl, 1 mM EDTA, and 1 mM 1,4-dithiothreitol (DTT). Based on a fact that theoretical pI of Mpro is 6.56, thus, all the following proteolytic kinetics assays were performed using this pH8.0 buffer. A standard curve was generated to convert the relative fluorescence unit (RFU) to the amount of the cleaved FRET substrate (pmol), and *k*_cat_ value was calculated using this plotted equation (Fig. 1c). Next, we characterized the proteolytic activity of purified Mpro by measuring the *K*_m_ and V_max_ values. When 0.25 µM Mpro was mixed with various concentrations of FRET substrate (0–45 µM), the initial velocity (V_I_) was measured separately to plot a reaction curve. Michaelis–Menten equation gave the best-fit values of *K*_m_ and V_max_ as 19.78 µM and 153.8 ΔRFU/s, respectively (Fig. 1d). The calculated *k*_cat_/*K*_m_ was 6990 s^−1^ M^−1^, which is slightly higher than the previously reported value of 3426.1 ± 416.9 s^−1^ M^−1^ [22], suggesting that the additional polyhistidine tag at the C terminus of Mpro is not detrimental to its proteolytic activity. This validation of the enzymatic activity of purified Mpro is crucial for the development of a reliable sandwich-like FP screening assay.

### General principle of a novel sandwich-like FP screening assay

Because of the inadequacy of existing HTS methods, we first developed a novel sandwich-like FP screening assay for the rapid discovery of antiviral agents targeting Mpro. FP technique has been widely utilized to identify antagonists targeting protein-protein interactions in drug discovery field [23]. In a FP screening assay, the change of millipolarization unit (mP) value is dependent on the molecular weight of fluorescent moiety provided that temperature and viscosity remain constant. For a small fluorescent tracer, the interaction with a macromolecule can be continuously monitored through the increase of mP value when the binding complex is formed. In this study, we first combined this FP technique with BAS to develop a novel and step-by-step sandwich-like FP screening assay to quickly identify inhibitors against Mpro. More importantly, this innovative FP screening assay is a simple, economical and practical strategy to rapidly screen large compound libraries for the discovery of novel Mpro inhibitors.

**Fig. 2:**
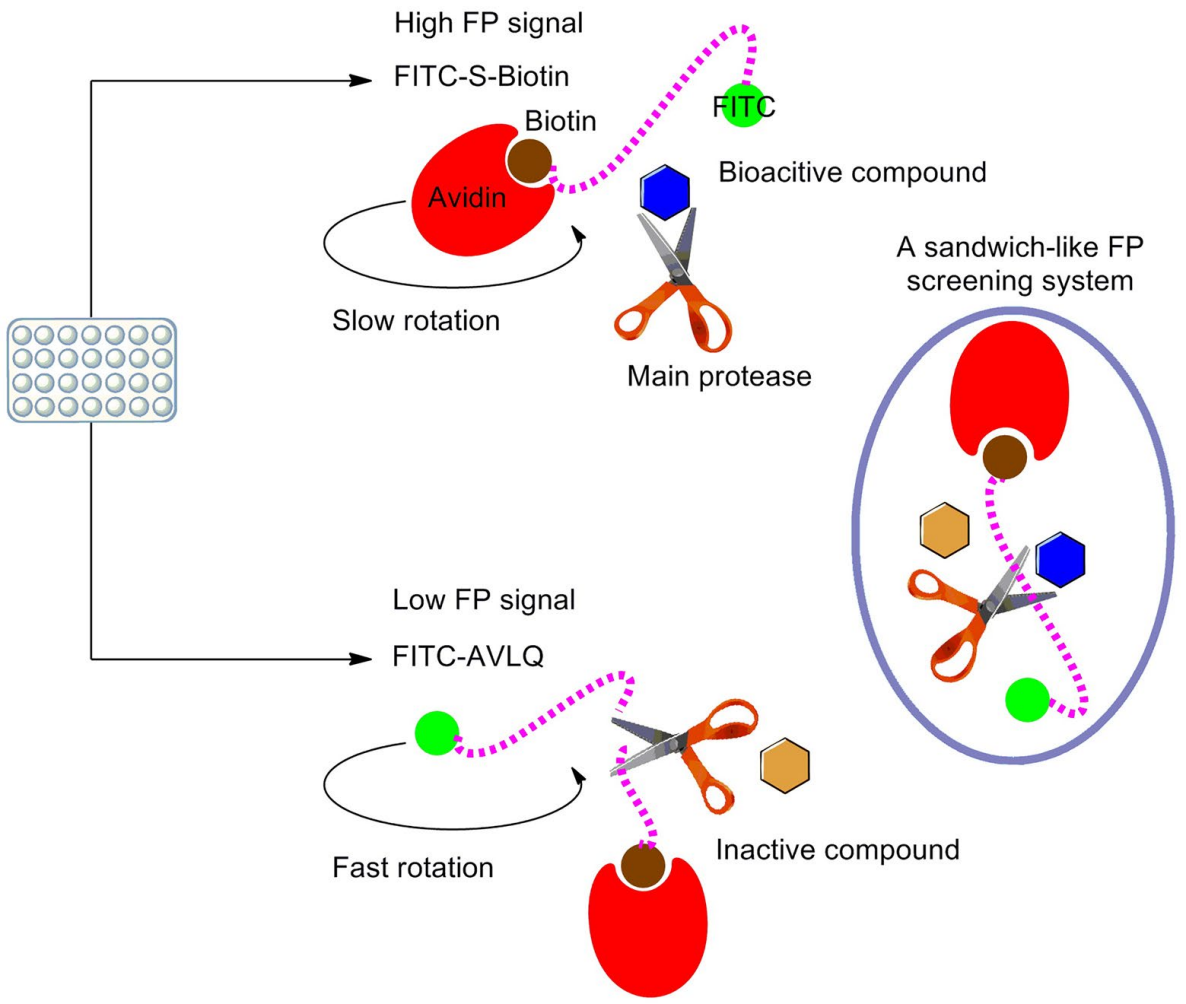
Schematic scenario for a sandwich-like FP screening assay principle. This first reported sandwich-like FP screening assay is based on the FP technique combining with BAS. Incubation of FP tracer (FITC-S-Biotin, purple dashed line) with Mpro (scissor) and subsequent addition of avidin (red crescent) produced a FP signal that was proportional to the relative amount of cleaved and uncleaved FP tracer. The uncleaved FP tracer produced a high mP value upon binding to avidin because of the exciting of bioactive compounds (blue hexagon), whereas the cleaved, a small FITC-AVLQ fragment that cannot bind to avidin produced a low mP value because of the exciting of inactive compounds (yellow hexagon). This newly developed screening protocol is mainly divided into three separate steps for laboratory use, and the appearance of this screening system looks like a sandwich

As previously described in a FRET assay, we synthesized a fluorescein isothiocyanate (FITC) and biotin dual-labeled small peptide as a FP tracer, FITC-AVLQSGFRKK-Biotin (FITC-S-Biotin), which was generated from previously used FRET substrate. Ideally, the highly active Mpro can adequately hydrolyze this FP tracer to yield two peptide fragments, FITC-AVLQ and SGFRKK-Biotin. This FITC labeled small peptide with smaller molecular weight emits light in all directions due to a fast rotation, resulting in a low FP signal. If the excited FP tracer is bound to a large protein avidin, it rotates slower due to the formation of a large binding complex. As a result, the emitting light remains in the same direction as that of the incident light, exhibiting a high FP signal. If bioactive compounds inhibit Mpro enzymatic activity, the excited uncleaved FP tracer is bound to a large binding partner avidin, and FITC-labeled complex has a higher molecular weight, resulting in a slow rotation and high mP value. In contrast, if the excited FP tracer is cleaved by Mpro to release a small FITC-AVLQ fragment, this cleaved small peptide fragment would rotate more rapidly, resulting in a low mP value due to the existing of inactive compounds (Fig. 2). Thus, any potential bioactive compounds targeting Mpro can be rapidly and easily identified by intensively monitoring the change of mP value in this sandwich-like FP screening assay.

### Optimization of a sandwich-like FP screening protocol for the discovery of SARS-CoV-2 Mpro inhibitors

**Fig. 3:**
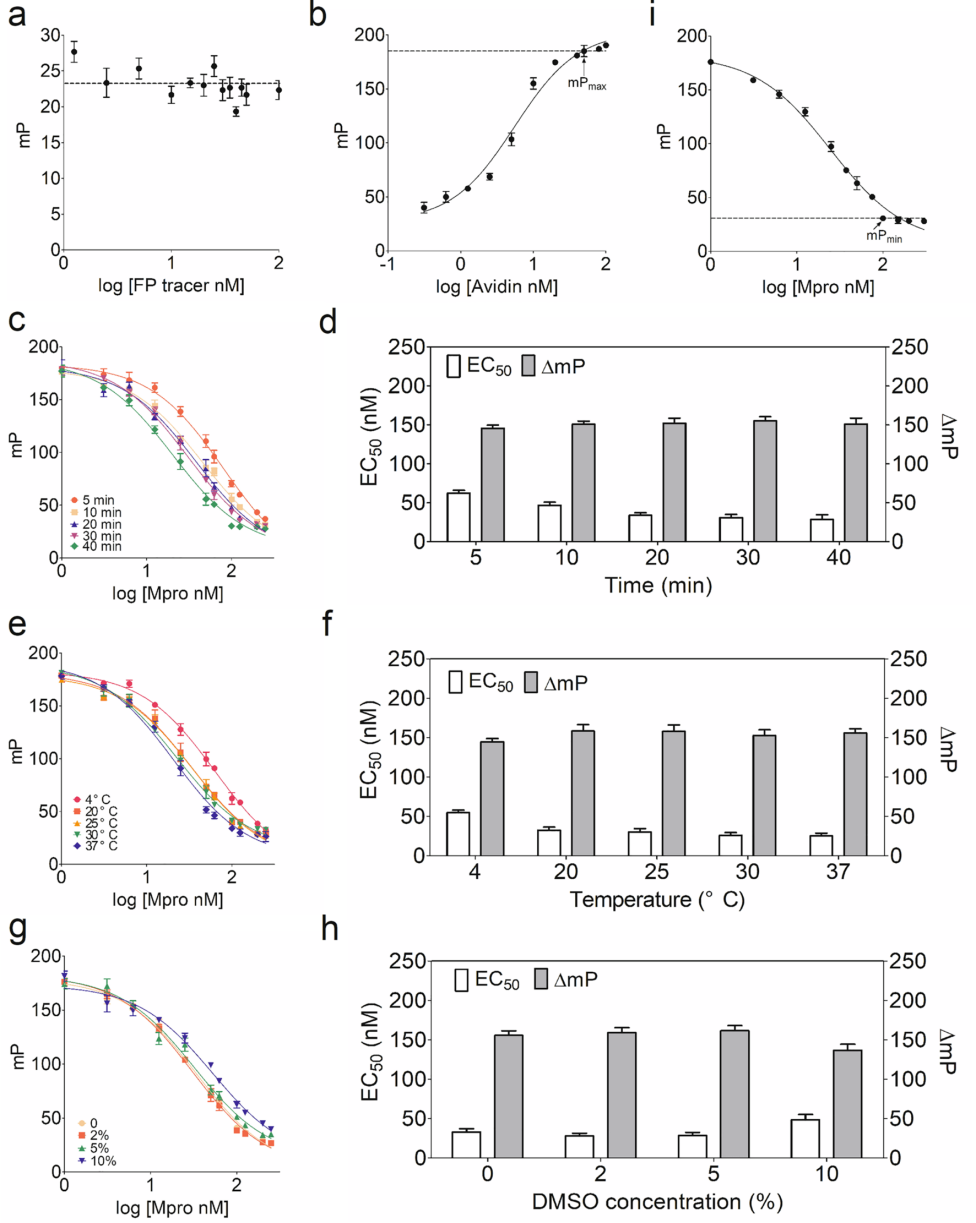
Optimization of the newly developed sandwich-like FP screening assay. **a** Determination of an optimal working concentration of FP tracer in a FP screening assay. After addition of the FP tracer dilution at an indicated concentration in a black 384-well microplate, the mP value was measured. The dashed line in the presented figure represents the average of mP values. **b** The binding curve between FP tracer and avidin. A fixed amount of FP tracer was incubated with avidin at the indicated concentrations for 5 min at RT, and then the mP value was monitored. The maximal mP value (mP_max_) indicated the arrival of the binding plateau, and an optimal amount of avidin was determined for screening. **c**, **d** The proteolytic reaction progression curves of Mpro at the indicated time. The mixture containing a fixed amount of FP tracer and increasing concentrations of Mpro was incubated at different time intervals. After quenching the proteolytic reaction by avidin, mP value was recorded to determine the EC_50_ and ΔmP values. **e**, **f** The proteolytic reaction progression curves of Mpro at the indicated temperature. The reaction mixture as described above was incubated for 20 min at the indicated temperature. After plotting the reaction curve according to the mP value, the EC_50_ and ΔmP values were compared separately to determine an optimal working temperature. **g**, **h** DMSO tolerance test in a FP screening assay. The proteolytic reaction curves of Mpro in the absence or presence of up to 10% DMSO were plotted. All experiments were independently repeated in triplicate. **i** Determination of an optimal concentration of Mpro at the optimized conditions in a FP screening assay. The reaction mixture was incubated for 20 min at RT, and then quenched by avidin. After measurement of mP value, the proteolytic reaction curve was plotted. The minimum mP value (mP_min_) indicated an endpoint of proteolytic reaction, which can be regarded as an optimal working concentration of Mpro used in this FP screening assay

As previously noted, we used FITC-AVLQSGFRKK-Biotin as the tracer in this FP screening assay, but the working concentration of the FP tracer can affect the screening assay sensitivity and cost. To select an optimal working concentration of the FP tracer, we examined the mP values of the FP tracer in various concentrations. A negligible variation of the mP signal was observed when FP tracer concentration was diluted below 100 nM. Importantly, the lowest concentration (10 nM) still provided a reliable mP signal. The mP signal was quite stable when the concentration of FP tracer was increased from 10 to 60 nM (Fig. 3a). Considering the significance of high sensitivity with the lowest background, we selected 20 nM of FP tracer as an optimal working concentration for this FP screening assay.

The activity of bioactive compound in this FP screening assay only relies on the binding of avidin to the FP tracer labeled with both FITC and biotin. Mpro could cleave the FP tracer to generate a small FITC-AVLQ fragment. The maximum mP (mP_max_) value was determined by titration of uncleaved FP tracer with avidin as shown in Fig. 3b. The mP_max_ value reached 180 with the addition of sufficient avidin, and it did not increase further after addition of more avidin. Based on the titration shown in Fig. 3b, we chose to quench this FP screening assay with 50 nM avidin. Obviously, this represented a 10-fold molar excess of tetrameric avidin in binding equivalence. The excess avidin used in this FP screening assay could efficiently quench the enzymatic reaction by making the FP tracer inaccessible to Mpro.

Because the proteolytic reaction of Mpro was typically time and temperature-dependent in this FP screening assay, an optimized incubation time was first determined at room temperature (RT). Surprisingly, the apparent half concentration of maximal effect (EC_50_) and dynamic range (ΔmP) values showed little changes from 20 to 40 min, indicating that a complete proteolytic reaction ends within 20 min (Fig. 3c, d). Based on the time course of the apparent EC_50_ values, the incubation time of Mpro proteolytic reaction process could be defined in 20 min in this FP screening assay. As illustrated in Fig. 3e and f, the changes of apparent EC_50_ and ΔmP values were quite stable after incubation at 20 °C, 25 °C, 30 and 37 °C for 20 min, respectively. Moreover, both mP values and proteolytic reaction curves were highly overlapped during this period of incubation. All these data strongly suggested that Mpro proteolytic reaction is significantly stable, and the reaction mixture should be incubated for 20 min at RT in the natural product screening.

The concentration of dimethyl sulfoxide (DMSO), a commonly used dissolvent, was also tested in this FP screening assay. The Mpro proteolytic reaction curves were well maintained even in the presence of up to 5% DMSO, and the apparent EC_50_ and ΔmP values also showed a stable dynamic range (Fig. 3g, h). These results suggested that the actual working concentration of DMSO used in this assay is less than 5% to achieve a reliable screening for natural products.

Another important question is what concentration of Mpro should be used in this FP screening assay because Mpro concentration has a decisive influence on the sensitivity of this screening assay. Using the optimized conditions mentioned above, a proteolytic course for the cleavage of the 20 nM FP tracer at the indicated Mpro concentrations was determined. In this proteolytic reaction, the cleaved FP tracer has a low mP value, but the uncleaved FP tracer has a high mP value. The result in Fig. 3i showed a significant decrease of mP value as more FP tracer is cleaved by Mpro. In the presence of 200 nM Mpro, the minimum mP (mP_min_) value appeared and yielded ΔmP value of 140 as the dynamic range, indicating the endpoint of Mpro proteolytic reaction. Based on this result, 200 nM of Mpro was selected for this FP screening assay to achieve a quite large dynamic range and high sensitivity.

### Evaluation of the sandwich-like FP screening assay by GC-376 and ***Z*** factor

**Fig. 4:**
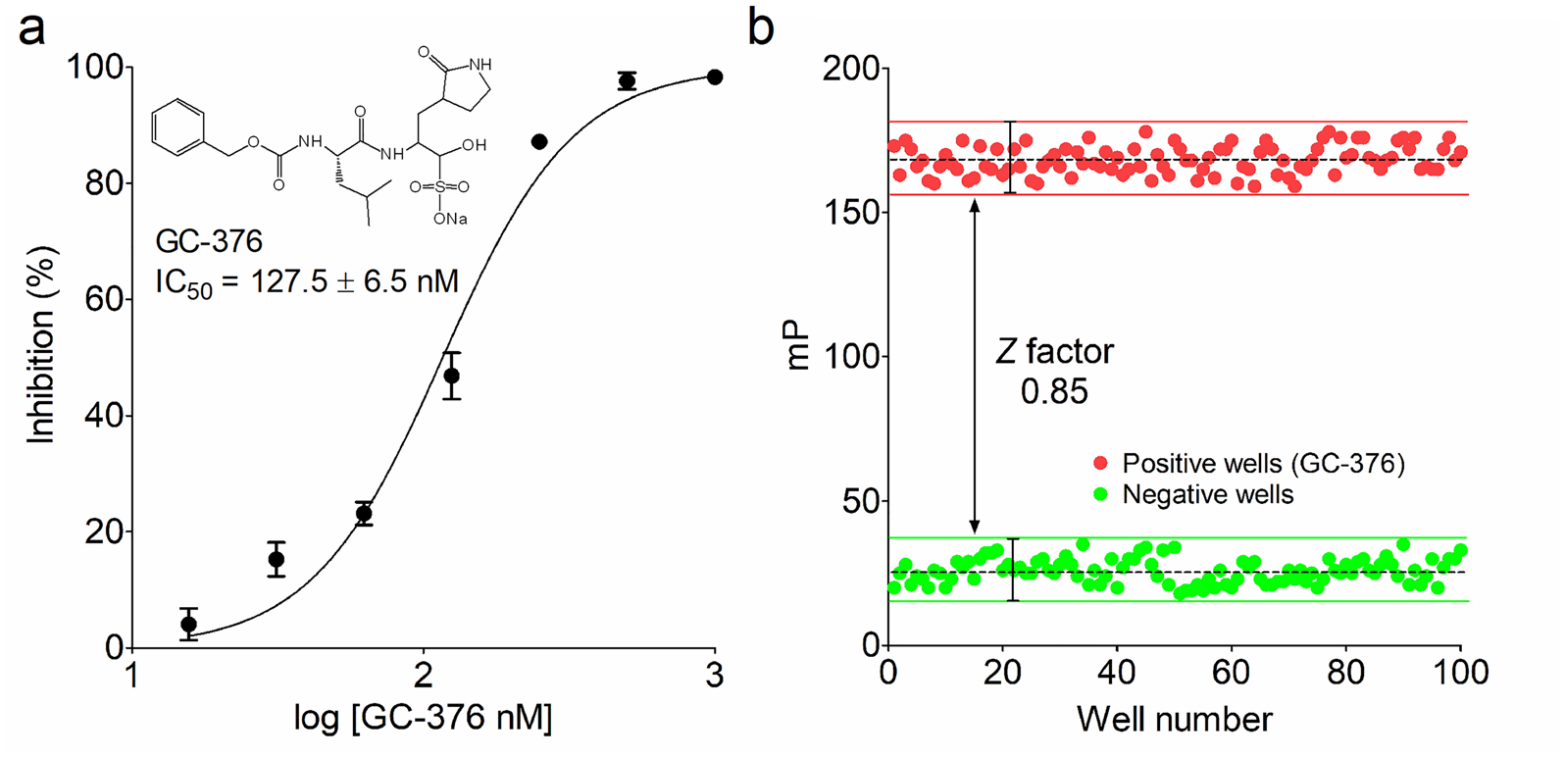
The quality assessment of the FP screening assay. **a** The inhibitory activity of GC-376 in a FP screening assay. Briefly, 30 µL mixture of 0.4 µM Mpro and GC-376 at the indicated concentrations was preincubated for 35 min at RT, and then 30 µL dilution of 40 nM FP tracer was added to initiate the proteolytic reaction. After addition of avidin, the mP value was measured to calculate the IC_50_ value of GC-376. Three independent experiments were performed. **b** Assessment of *Z* factor of the FP screening assay. GC-376 was used as a positive control, and the measured mP value from negative and positive wells were shown, respectively. The *Z* factor of this FP screening assay was 0.85, and a high signal window was represented by a black straight line. The experiment was performed in triplicate

GC-376, a commonly used covalent pancoronavirus inhibitor against Mpro, has been widely used to verify the sensitivity and accuracy of a newly developed screening assay for the discovery of Mpro inhibitors [11, 13, 14]. As expected, GC-376 exhibited a half-maximum inhibitory concentration (IC_50_) value of 127.5±6.5 nM in this FP screening assay (Fig. 4a). Our observed inhibitory activity of GC-376 was highly consistent with the published reports in a FRET screening assay [24, 25]. Together, the result from this experiment adequately demonstrated the technique feasibility and reliability of this FP screening assay for rapid discovery of Mpro inhibitors.

The statistical parameter *Z* factor, which is commonly used to assess the total quality of a HTS method, was determined in a black 384-well microplate. In general, only HTS assays with *Z* factor of 0.5 or higher are suitable for screening, and the assays with smaller *Z* factor are required to be further optimized [23]. To assess this FP screening assay, 1 µM of GC-376 was used to serve as a positive control for quality assessment. As shown in Fig. 4b, the *Z* factor of this FP screening assay was 0.85, indicating that this assay is robust and amenable to HTS.

### Dieckol is a novel competitive inhibitor against SARS-CoV-2 main protease

**Fig. 5:**
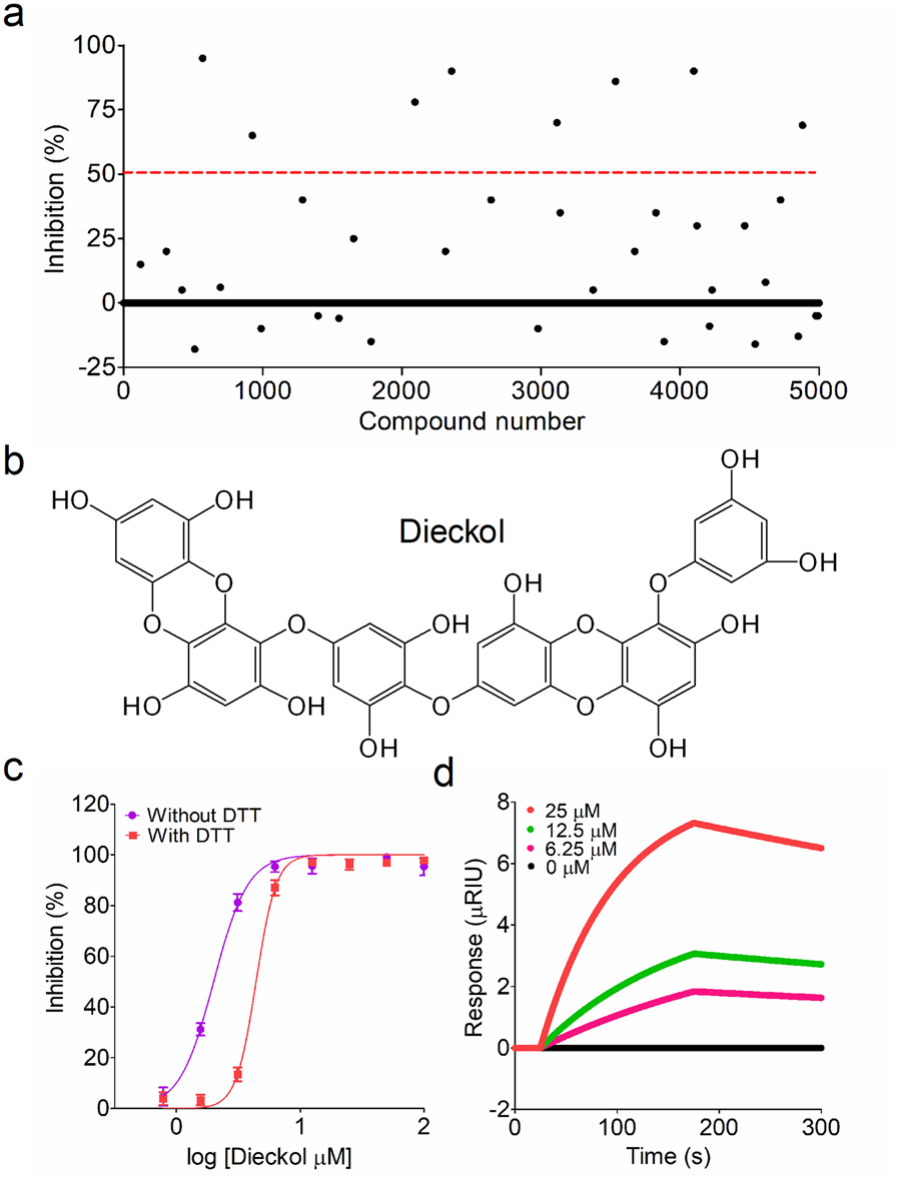
The primary screening of a natural product library. **a** A general view for the primary screening. The red dashed line in the figure indicated 50% inhibition, and the inhibitory activity of candidate compound was higher than this defined red line. **b** The chemical structure of dieckol, a natural phlorotannin component extracted from *Ecklonia cava*. **c** The inhibitory activity of dieckol in the FP screening assay. The inhibitory analysis of dieckol in the absence or presence of 1 mM DTT was performed as described for GC-376. The IC_50_ value of dieckol was analyzed using GraphPad Prism 5.0. All assays were performed in triplicate. **d** Analysis of interaction between dieckol and Mpro using the SPR assay. The binding kinetics of dieckol (0, 6.25, 12.5, 25 µM) to immobilized Mpro was plotted according to the change of RIU value

In the primary screening, a library of 5000 natural products was screened for the rapid discovery of Mpro inhibitors using an optimized FP screening assay. Considering the stringent caution of DTT addition for the accuracy in candidate compound screening [26], all the FP screening assays were performed in the presence of 1 mM DTT. This primary screening identified 8 compounds that exhibited IC_50_ values of  >50% at the screening concentration (Fig. 5a). Subsequently, a dose-response curve analysis showed that dieckol is a novel SARS-CoV-2 Mpro inhibitor, with an IC_50_ value of 4.5 ± 0.4 µM (Fig. 5b, c). More interestingly, dieckol also exhibited a strong inhibition against Mpro with an IC_50_ value of 2.9 ± 0.2 µM in the absence of DTT (Fig. 5c), and the inhibitory activity of dieckol was not significantly affected by addition of DTT in a FP screening assay.

Surface plasmon resonance (SPR) technology has been widely used in assessing the binding affinity between bioactive compound and target protein, and has been regarded as a conventional biochemical method in the analysis of analyte binding affinity [23, 27]. The real-time SPR binding kinetics showed that dieckol significantly increased the real refractive index unit (RIU) response in a dose-dependent manner, and the dissociation constant (*K*_D_) was recorded as 0.22 µM, indicating a strong interaction between dieckol and Mpro in vitro (Fig. 5d). Moreover, dieckol exhibited a clear feature of fast-associating (*k*_a _= 1.02 × 10^−3^/M s) and slow dissociating (*k*_d _= 2.26 × 10^−4^/s) inhibition mode, suggesting a strong target occupancy.

**Fig. 6:**
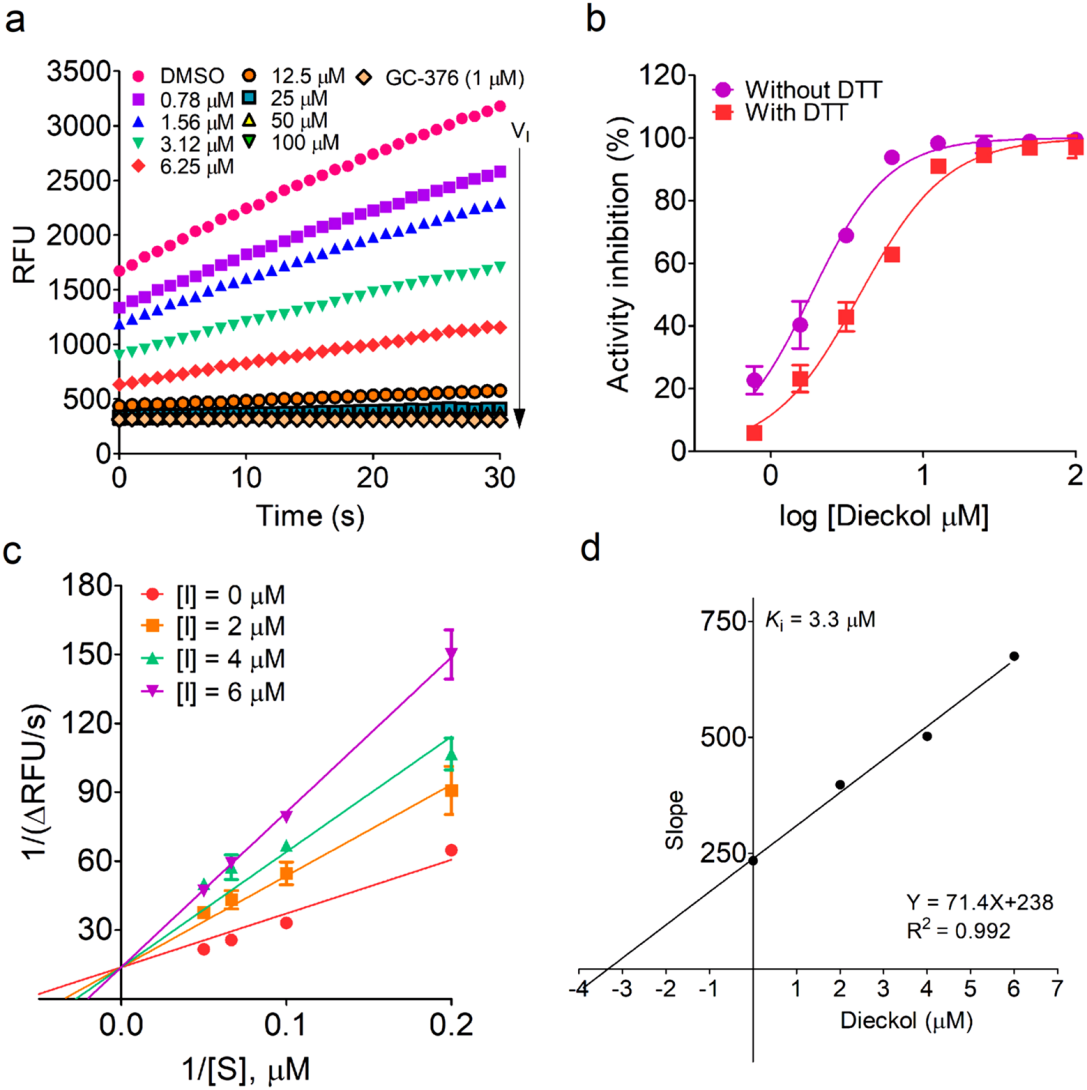
The inhibition mechanism of dieckol. **a** The inhibitory activity of dieckol in the FRET assay. Mpro (0.4 µM) was preincubated with the indicated concentrations of dieckol at RT for 35 min in the presence of 1 mM DTT in FP assay buffer. After addition of FRET substrate, the RFU value was separately measured every second for 3 min by a microplate reader (BioTek). The V_I_ was calculated by the slope of a linear regression in the first 30 s. DMSO and GC-376 (1 µM) was used as the negative and positive control, respectively. The RFU value was notably quenched by dieckol in a dose-dependent manner. **b** The inhibitory activity of dieckol in the absence or present of DTT. The IC_50_ values were separately obtained from dose-response curves by plotting the V_I_ against various concentrations of dieckol in GraphPad Prmis 5.0. **c** The Lineweaver–Burk plots for analysis the inhibition mechanism of dieckol on Mpro using a FRET assay. **d** The secondary plots for a *K*_i_ value of dieckol in the FRET substrate

To interrogate the inhibition mechanism of dieckol against Mpro, we performed various enzyme kinetics studies with the indicated concentrations of dieckol using a FRET assay. As shown in Fig. 6a, dieckol exhibited a significant dose-dependent inhibitory activity against Mpro with an IC_50_ value of 3.8 ± 0.3 µM in a FRET assay. Interestingly, the absence of DTT did not notably alter this inhibitory curve, and the measured IC_50_ value was 1.8 ± 0.2 µM (Fig. 6b). These results were consistent with the observations in a FP screening assay. Next, these obtained inhibition data of dieckol were analyzed using Lineweaver–Burk (LB) plot graphical method to determine the possible kinetic model. Notably, subsequent LB plot yielded a series of straight lines with the same y-axis intercept in the presence of dieckol at the indicated concentrations, suggesting that dieckol is a competitive inhibitor against Mpro with an inhibitory constant (*K*_i_) value of 3.3 µM, because of an obvious character of the same V_max_ but varying *K*_m_ values in the presence of dieckol at different concentrations (Fig. 6c, d).

**Fig. 7:**
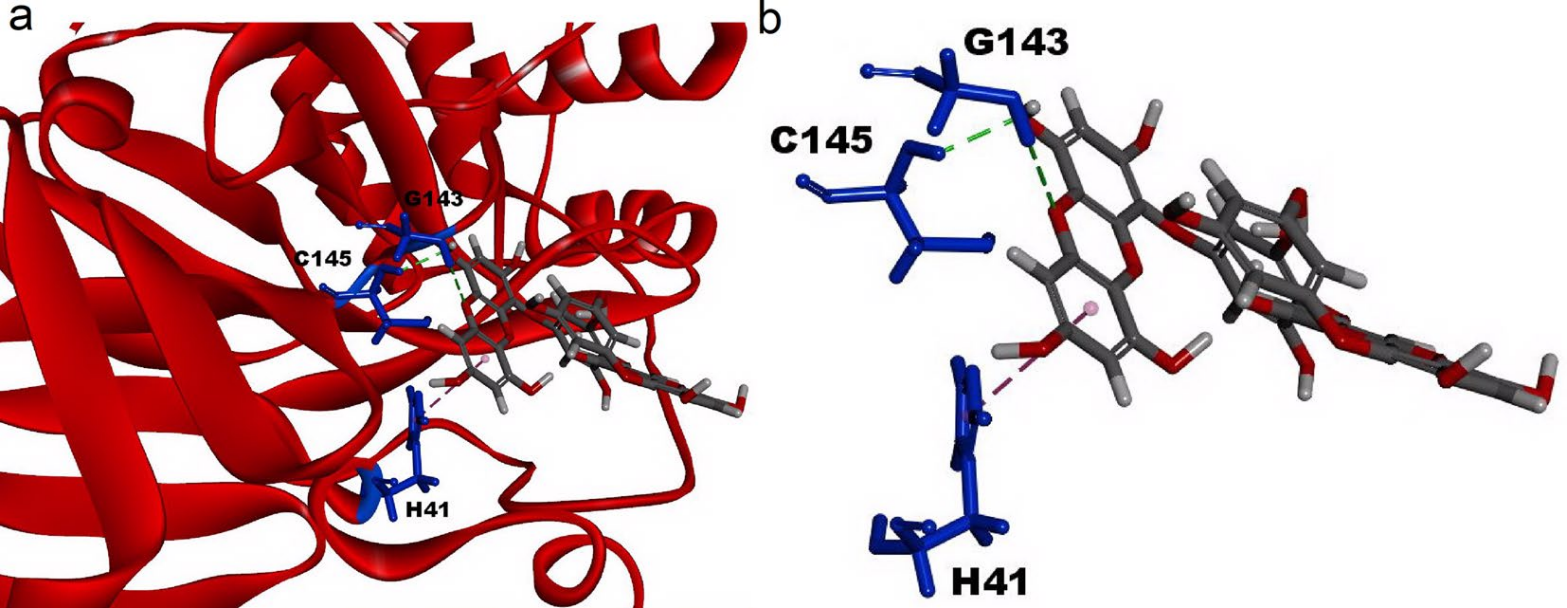
The predicted molecular docking mode of dieckol in the catalytic sites of Mpro crystal structure. **a** A molecular docking model between dieckol and the catalytic sites of Mpro. The blue sticks represent the functional amino acids interacting with dieckol, which are exhibited by a stick model. **b** A detailed illustration for dieckol binding to the catalytic sites of Mpro. The residues of Cys145 and Gly143 formed strong hydrogen-bond interactions highlighted by the green dashed lines with the hydroxyl group and the oxygen atom of dieckol, respectively. A purple dashed line indicated a Pi-Pi interaction between His41 and benzene ring of dieckol

Next, a computational docking stimulation was performed to further investigate the inhibition mechanism of dieckol. The reported crystal structure of Mpro in complex with MI-23 (PDB code: 7D3I) was retrieved as the dieckol docking target [28]. As shown in Fig. 7a and b, the hydroxyl group of dieckol formed a conventional hydrogen bond with Cys145 (2.24 Å) and a Pi-Pi interaction (4.59 Å) with His41, which are the catalytic residues of Mpro. Additionally, the hydroxyl group of dieckol formed another hydrogen bond with Gly143 (2.74 Å) for further anchoring dieckol to the catalytic sites of Mpro. This simulation result showed that dieckol has a C-Docking score value of 82.75. This predicted docking model suggested that dieckol binds to the catalytic sites of Mpro through strong hydrogen-bond interactions, which supports a competitive inhibition mechanism of dieckol against Mpro.

## Discussion

The current COVID-19 pandemic has imposed a huge and unprecedented global healthcare crisis. Researchers around the world have made extensive efforts to develop effective vaccines and antiviral agents to fight against COVID-19. However, no efficacious antiviral agents are currently available to prevent or treat SARS-CoV-2 infection. The genetic reshuffling, mutations, and interspecies transmission of the RNA viruses highlight the urgent need for the development of broad-spectrum antiviral drugs. The highly conserved Mpro is responsible for the viral polyprotein proteolytic process and genomic RNA replication. Thus, Mpro has been considered to be a promising therapeutic target for the development of antiviral agents [6–8]. Currently, the screening and characterization of novel Mpro inhibitors using a rapid, simple and miniaturized HTS assay are of crucial importance [21].

The homogenous and highly sensitive FP technique has been widely used in drug discovery, analytical biochemistry, measurement of biomolecular activity and disease diagnosis [23, 29–31]. This powerful approach is based on a fact that a positive alteration in apparent molecular weight of a fluorescent moiety in solution is indicated by an increasing change of mP value in a FP assay. In this study, we have developed a simple and miniaturized screening assay for rapid discovery of Mpro inhibitors using this FP technique combined with BAS. The decrease of mP value is caused by release of a small FITC-AVLQ fragment after the cleavage of FP tracer by Mpro, but bioactive compound may strongly inhibit this proteolytic activity. As a result, the inhibitory activity of bioactive compound produces an increased mP value because of the formation of a high molecular weight of avidin-tracer complex. Hence, validation of bioactive compound can be conveniently achieved by directly measuring the increase of mP value. In this sandwich-like FP screening assay, a small FP tracer modified by a FITC moiety on the N-terminus and a biotin group on the C-terminus is derived from Mpro natural cleavage site, and this similar peptide was also used in a reported FRET assay [7]. Moreover, this FP tracer can be cleaved efficiently by active Mpro prepared in *E. coli* cells. To further improve the reliability and sensitivity, various biochemical conditions including the concentration of FP tracer, incubation time, temperature, DMSO tolerance, and the concentration of Mpro were systematically optimized in this FP screening assay. Subsequently, a high-quality FP screening assay with a high *Z* factor (0.85) was successfully developed. Furthermore, GC-376, a covalent Mpro inhibitor identified by a FRET screening assay, exhibits a strong inhibitory activity in this developed approach, and this result consists with the literature reports [24, 25]. Therefore, this FP screening assay shows a high selectivity and sensitivity. Unlike a FRET assay reported previously, the FP screening assay developed in this research is more homogeneous, rapid, robust, and economical. Because only a small amount FP tracer is used (20 nM/well) in this protocol and it takes less than one hour to finish each screening cycle, this FP screening system is very cheap, simple and rapid, which is ideal for a large-scale screening. Moreover, this FP screening assay directly monitors the endpoint change of mP value rather than relative quenching ratio in a FRET screening system. Considering this biochemical feature, the FP screening assay is more reproducible. Furthermore, the effect of potential fluorescent interferants is minimized because the fluorophore, FITC, used in this system has a high quantum yield for emission at 535 nm wavelength, which is outside the range of most fluorescent molecules in natural products.

Using this innovative FP screening assay, a pilot screening of natural product library was performed to identify potent inhibitors targeting Mpro. As a result, dieckol exhibited a dose-dependent inhibitory activity against Mpro in this screening assay. Moreover, dieckol showed a similar inhibitory activity in a FRET kinetics assay with a *K*_i_ value of 3.3 µM. Dieckol is a major natural phlorotannin ingredient extracted from a Chinese traditional medicine *Ecklonia cava*, which was documented in Compendium of Materia Medica published in Chinese Ming dynasty. *Ecklonia cava* is widely used as a folk medicine for the treatment of goiter, scrofula, urinary diseases, anti-asthmatic, and postpartum women [32]. As a natural component, dieckol shows a broad range of bioactivities, including antibacterial, anticancer, antioxidant, antidiabetic, and other medicinal applications [33]. Recent studies have shown that dieckol exhibits antiviral activity by inhibiting HIV reverse trancriptase, SARS-CoV main protease and influenza A virus neuraminidase [34–36]. A recent molecular dynamics simulation has predicted that dieckol might be a potential antiviral agent targeting RBD-ACE2 interaction [37], but more solid biochemical evidence for its antiviral mechanism is urgently needed [38]. Unlike the previously reported covalent and peptidomimetic inhibitors, our systematic biochemical studies warrant that dieckol is a novel competitive inhibitor against Mpro in vitro. However, more in vivo experiments will be required for further validation.

## Conclusions

Overall, we combined the FP technique with BAS for the first time to develop a novel sandwich-like FP screening assay for rapid discovery of Mpro inhibitors. Importantly, from a natural product library, dieckol is identified as a novel competitive inhibitor against Mpro in vitro. This newly developed FP screening assay can be easily generalized for rapid large-scale screening of antiviral agents targeting viral proteases. As a promising lead compound, dieckol provides an excellent starting point to generate more potent and selective antiviral agents targeting Mpro.

## Methods

### Chemical reagents

The natural product library, GC-376, and 7-methoxycoumarin-4-acetic acid (MCA) were purchased from TargetMol (Shanghai, China), and the purity was more than 97.0% in mass spectrometry analysis. Dieckol was commercially provided by Yuanye Biotech (Shanghai, China). All tested compounds were dissolved in DMSO at a final concentration of 20 mM, and stored at − 20 °C before use. The FRET fluorogenic substrate (MCA-AVLQSGFR-Lys(Dnp)-Lys-NH_2_), FP tracer (FITC-AVLQSGFRKK-Biotin) and FITC-AVLQ peptide were chemically synthesized (GL Biochem Shanghai Co. Ltd., Shanghai, China), and the purity was more than 95.0%.

### Expression and purification of SARS-CoV-2 main protease

SARS-CoV-2 main protease (Mpro) was prepared as previously described in reported publications [39, 40]. Briefly, a codon-optimized DNA sequence encoding SARS-CoV-2 Mpro (GenBank: YP_009725301.1) was chemically synthesized by Gene Universal Biotech (Chuzhou, China), and then cloned into a pET-21a(+) vector with *Nde*I and *Xho*I restriction sites. The N-terminal methionine is removed by *E. coli* methionine aminopeptidase, and there are extra LEHHHHHH residues at the C terminus.

For Mpro purification, the soluble Mpro was expressed in *E. coli* Rosetta (DE3) cells after induction with 0.2 mM IPTG at 30 °C for 8 h. The induced *E. coli* cells were harvested and resuspended in the lysis buffer (50 mM Tris, 150 mM NaCl, pH8.0) for sonication. After removing the cell debris by centrifugation, the supernatant was purified by an affinity chromatography method using a HisTrap^TM^ chelating column (Cytiva, Uppsala, Sweden), and then the purity of purified Mpro was further analyzed by SDS-PAGE. Subsequently, all fractions were dialyzed overnight in TBS buffer (25 mM Tris, 150 mM NaCl, 1 mM EDTA, 1 mM DTT, pH8.0) and stored at − 80 °C before use.

### MCA standard curve

A MCA standard curve was plotted as previously described [39]. MCA was diluted to 500, 250, 125, 62.5, 31.25 pmol using the FP buffer (10 mM Tris, 50 mM NaCl, 1 mM EDTA, 1 mM DTT, pH8.0), and then added to a black 384-well microplate (PerkinElmer, Waltham, USA) in triplicate. The relative fluorescence units (RFU) values were measured with an excitation wavelength of 320 nm and emission wavelength of 405 nm at 25 °C using a microplate reader (BioTek, Winooski, USA). The RFU signal was plotted against total MCA amount (pmol) to produce a linear curve and an equation.

### Enzymatic kinetics of SARS-CoV-2 main protease

As previously described, the enzymatic kinetics was determined using a FRET assay [39]. For reaction buffer optimization, the mixture containing 0.25 µM Mpro and 10 µM FRET substrate in various pH values was added to a black 384-well microplate. Subsequently, the change of RFU value was continuously recorded every second for 3 min by a microplate reader with an excitation wavelength of 320 nm and emission wavelength of 405 nm at room temperature (RT). The initial velocity (V_I_) of the proteolytic activity was calculated by a linear regression for the first 30 s of the kinetic progress curve plotted in GraphPad Prism 5.0. Experiments were performed in triplicate. All the following enzymatic assays were carried out in pH8.0 buffer, because Mpro displays the highest proteolytic activity in pH8.0 buffer. These used reaction buffers are listed as below: buffer #1 (10 mM MES, 50 mM NaCl, 1 mM EDTA, 1mM DTT, pH6.0); buffer #2 (10 mM HEPES, 50 mM NaCl, 1 mM EDTA, 1mM DTT, pH7.0); buffer #3 (FP assay buffer: 10 mM Tris, 50 mM NaCl, 1 mM EDTA, 1 mM DTT, pH8.0).

For the measurement of *K*_m_ and *k*_cat_ values, a FRET substrate was added into the Mpro solution (0.25 µM) to generate 8 final concentrations ranking from 1.25 to 45 µM in 50 µL of FP assay buffer. The proteolytic reaction progression was immediately monitored every second for 3 min by a microplate reader, and the V_I_ was calculated for the first 30 s by a slope of a linear regression. The V_I_ was plotted against the FRET substrate concentration using a Michaelis–Menten equation in GraphPad Prism 5.0. The *K*_m_ and *k*_cat_ values were calculated using this fitted equation and MCA standard curve.

### An optimization procedure for a sandwich-like FP screening assay

The measurement for the optimal amount of the used FP tracer in this FP screening assay followed the published protocol [23]. Briefly, the FP tracer (2 mM) was diluted to the fixed concentrations ranking from 1.25 to 100 nM in the FP assay buffer, and 60 µL of the dilutions were pipetted into a black 384-well microplate in triplicate. After incubation for 15 min at RT, the mP value was recorded by a microplate reader with the excitation at 485 nm and emission at 535 nm.

To determine the optimal concentration of avidin (Sigma-Aldrich, St. Louis, USA) in this FP screening assay, 40 nM FP tracer (30 µL/well) was mixed thoroughly with 30 µL of the avidin dilutions (0∼200 nM, 30 µL/well) in a black 384-well microplate in triplicate. The reaction mixture was incubated for 5 min at RT, and then the measurement of mP value was followed as described above.

For the FP screening assay stability test, 40 nM FP tracer (30 µL/well) was gently mixed with the increasing concentrations of Mpro dilutions (0∼500 nM, 20 µL/well) in a black 384-well microplate in triplicate, and incubated for 5, 10, 20, 30, 40 min at RT, respectively. After addition of avidin (300 nM, 10 µL/well), the reaction mixture was further incubated for 5 min at RT, and then the mP value was separately recorded by a microplate reader.

As described above, the reaction mixture containing 40 nM FP tracer (30 µL/well) and Mpro dilutions (0∼500 nM, 20 µL/well) was incubated for 20 min at 4, 20, 25, 30, 37 °C, respectively. After quenching the proteolytic reaction by avidin, the mP value was measured separately.

For DMSO tolerance assay, varied concentrations of DMSO (from 0 to 10%) were added into the mixture containing 40 nM FP tracer (30 µL/well) and Mpro dilutions (0∼500 nM, 20 µL/well), and the reaction mixture was incubated for 20 min at RT. After quenching by avidin for additional 5 min, the mP value was monitored as described above. All the proteolytic reaction curves of Mpro in previously  mentioned experiments were plotted, and the EC_50_ and dynamic range (ΔmP) values were calculated using these fitted curves plotted by GraphPad Prism 5.0. All mentioned assays were independently performed in triplicate.

As mentioned above, the reaction mixture containing 40 nM FP tracer (30 µL/well) and Mpro dilutions (0∼500 nM, 20 µL/well) was incubated for 20 min at RT. The mP value was measured after additional 5 min incubation of avidin. The proteolytic reaction curve of Mpro was plotted using GraphPad Prism 5.0. An optimal working concentration of Mpro should be regarded as a minimum amount of Mpro, which can adequately cleave the FP tracer into a small FITC-AVLQ fragment in the FP screening assay.

### Inhibitory activity of GC-376 in a sandwich-like FP screening assay

In all, 30 µL sample of 400 nM Mpro was incubated with various concentrations of GC-376 (8 two-fold dilutions; initial concentration: 2 µM) for 35 min at RT in a black 384-well microplate. Later, 20 µL sample of 60 nM FP tracer was added and incubated for 20 min at RT. After incubation of avidin for 5 min, the measurement of mP value was performed by following the previous protocol, and the inhibitory curve of GC-376 was plotted using GraphPad Prism 5.0. In this inhibitory activity test, the FITC-AVLQ peptide-free well was used as a negative control, and the well containing FITC tracer/avidin binding complex was used as a positive control. Three independent assays were performed. The IC_50_ value of GC-376 was calculated using the following equation:

1 $${\text{GC-376 inhibition}}\, (\%) = \frac{{\upmu }_{\text{Hit}}-{\upmu}_{\text{N}}}{ {\upmu }_{\text{P}}-{\upmu}_{\text{N}}} \times 100\%$$

where µ_Hit_, µ_N_ and µ_P_ represent the average mP values of the tested inhibitor, negative control, and positive control, respectively.

### Determination of *Z* factor

For the determination of *Z* factor, 30 µL sample of 400 nM Mpro and 1 µM GC-376 (positive control) or DMSO (negative control) was incubated for 35 min at RT, respectively. Each control contained 100 wells in a black 384-well microplate. As described above, the measurement of mP value was performed using a microplate reader. The *Z* factor was calculated according to following equation [23]:

2 $$Z=1-\frac{{3\times (\text{S}\text{D}}_{\text{N}}+{\text{S}\text{D}}_{\text{P}})}{\left|{{\upmu }}_{\text{N}}-{{\upmu }}_{\text{P}}\right|}$$

where µ_N_ and µ_P_ are the average of mP values obtained from the negative and positive controls, respectively. SD_N_ and SD_P_ are the standard deviations.

### The primary screening protocol of a natural product library

In this FP screening assay, 29 µL sample of 400 nM Mpro diluted in the FP assay buffer was mixed with 1 µL of natural product (1 mg/mL in DMSO) in a black 384-well microplate, and the mixture was further incubated for 35 min at RT before adding 20 µL sample of 60 nM FP tracer. After proceeding for 20 min at RT, the reaction was quenched by addition of 10 µL sample of 300 nM avidin, and the mP values were measured by a microplate reader. In each assay plate, GC-376 (1 µM) and DMSO were used as a positive and negative control, respectively. The well containing only 60 µL sample of 20 nM FITC-AVLQ peptide was used to assess the background noise. The inhibitory activity of screening compound was calculated using Eq. (1), and the candidate compounds (> 50% inhibition) were analyzed in triplicate to generate the dose-response curves in the second screening.

The inhibitory activity of dieckol in this FP screening assay was carried out as mentioned above. The initial concentration of dieckol was 100 µM, and 8 two-fold dilutions were prepared for the determination of IC_50_ value. To remove the DTT effect on the enzymatic assay [26], the IC_50_ value was also obtained in the absence of DTT in a FP screening assay.

### SPR binding assay

The binding affinity between dieckol and Mpro was measured using a real-time SPR spectroscopy instrument (Reichert 2SPR, Buffalo, USA). In all, 20 µL Mpro (1.6 mg/mL) in 10 mM NaAc buffer (pH5.5) was immobilized to a surface activated CM5 gold biosensor (Reichert Inc., New York, USA), and the active sites were quenched with 1 M ethanolamine buffer (pH8.0). After washing the sample loop, an indicated concentration of dieckol (0, 6.25, 12.5, 25 µM) was separately injected on the surface of the Mpro-captured biosensor at RT. The binding time of the analyte to the ligand was 90 s, and the dissociation kinetic was recorded during 330 s. Finally, the real RIU was continuously monitored, and the RIU can be related to arbitrary resonance unit (RU) as 1 µRIU = 0.733 RU. The *K*_D_ value was calculated using the analyte binding kinetic curve plotted by Trace Drawer software (Ridgeview Instruments, Sweden).

### Inhibition mechanism of dieckol against SARA-CoV-2 main protease

As described previously, the inhibitory activity of dieckol against Mpro was preformed using a FRET assay [11, 24]. In brief, 50 µL sample of Mpro (0.4 µM) was preincubated with the indicated concentrations of dieckol (initial concentration: 100 µM, 8 two-fold dilutions) at RT for 35 min in FP assay buffer, and the reaction was initiated by adding 5 µM FRET substrate. DMSO and GC-376 (1 µM) was used as a negative and positive control, respectively. The RFU change was separately measured as described previously every second for 3 min by a microplate reader. The V_I_ was calculated by a slope of the linear regression in the first 30 s, and the IC_50_ value was calculated using the following equation:

3 $$\text{D}\text{i}\text{e}\text{c}\text{k}\text{o}\text{l} \ {i}\text{n}\text{h}\text{i}\text{b}\text{i}\text{t}\text{i}\text{o}\text{n} \,(\%)=1-\frac{{\text{V}}_{\text{I} \left(\text{D}\text{i}\text{e}\text{c}\text{k}\text{o}\text{l}\right)}}{{\text{V}}_{\text{I} \left(\text{D}\text{M}\text{S}\text{O}\right)}} \times 100\%$$

All experiments were performed in triplicates.

To clarify the inhibitory mechanism of dieckol, the fixed concentrations of dieckol (0, 2, 4, 6 µM) were separately added to 50 µL sample of Mpro (0.4 µM), and the mixture was preincubated for 10 min at RT. After adding 5 µM FRET substrate, the V_I_ was measured using a FRET assay. The inhibition mechanism of dieckol was inferred using the LB plot and a *K*_i_ value was generated by its secondary plot [35, 41, 42].

### Molecular docking study

Molecular docking analysis was performed using BIOVIA Discovery Studio 2018R2 software (Accelrys, San Diego, USA), and the reported crystal structure of Mpro in complex with MI-23 (PDB code: 7D3I) was retrieved as the dieckol docking target [28]. After removing the water molecules, a possible binding pocket of dieckol in Mpro was defined according to the hot spots published previously to identify the potential hydrogen-bond interactions between the catalytic sites of Mpro and dieckol [7, 28]. The optimized docking result was generated and processed using C-DOCKER program.

## Data Availability

All data generated or analyzed during this study are included in this article.

## Abbreviations

BAS: Biotin-avidin system
COVID-19: Coronavirus disease 2019
DTT: Dithiothreitol
Dnp: 2,4-dinitropheno
FITC: Fluorescein isothiocyanate
FP: Fluorescence polarization
FRET: Fluorescence resonance energy transfer
HTS: High-throughput screening
MCA: 7-methoxycoumarin-4-acetic acid
mP: Millipolarization unit
Mpro: Main protease
RIU: Refractive index unit
RFU: Relative fluorescence unit
SARS-CoV-2: Severe acute respiratory syndrome coronavirus-2
SDS-PAGE: Sodium dodecyl sulfate polyacrylamide gel electrophoresis
SPR: Surface plasmon resonance

## References

[1] Hsu J (2020). Covid-19: What now for remdesivir?. BMJ..

[2] Dyer O (2020). Covid-19: Remdesivir has little or no impact on survival, WHO trial shows. BMJ.

[3] Brant A, Tian W, Majerciak V, Yang W, Zheng Z (2021). SARS-CoV-2: from its discovery to genome structure, transcription, and replication. Cell Biosci.

[4] Shang J, Wan Y, Luo C, Ye G, Geng Q, Auerbach A, Li F (2020). Cell entry mechanisms of SARS-CoV-2. Proc Natl Acad Sci U S A.

[5] Medina-Enríquez MM, Lopez-León S, Carlos-Escalante JA, Aponte-Torres Z, Cuapio A, Wegman-Ostrosky T (2020). ACE2: the molecular doorway to SARS-CoV-2. Cell Biosci.

[6] Goyal B, Goyal D (2020). Targeting the Dimerization of the Main Protease of Coronaviruses: A Potential Broad-Spectrum Therapeutic Strategy. ACS Comb Sci.

[7] Jin Z, Du X, Xu Y, Deng Y, Liu M, Zhao Y, Zhang B, Li X, Zhang L, Peng C, Duan Y, Yu J, Wang L, Yang K, Liu F, Jiang R, Yang X, You T, Liu X, Yang X, Bai F, Liu H, Liu X, Guddat LW, Xu W, Xiao G, Qin C, Shi Z, Jiang H, Rao Z, Yang H (2020). Structure of M^pro^ from SARS-CoV-2 and discovery of its inhibitors. Nature.

[8] Jin Z, Wang H, Duan Y, Yang H (2021). The main protease and RNA-dependent RNA polymerase are two prime targets for SARS-CoV-2. Biochem Biophys Res Commun.

[9] Li Z, Li X, Huang YY, Wu Y, Liu R, Zhou L, Lin Y, Wu D, Zhang L, Liu H, Xu X, Yu K, Zhang Y, Cui J, Zhan CG, Wang X, Luo HB (2020). Identify potent SARS-CoV-2 main protease inhibitors via accelerated free energy perturbation-based virtual screening of existing drugs. Proc Natl Acad Sci U S A.

[10] Chen Z, Cui Q, Cooper L, Zhang P, Lee H, Chen Z, Wang Y, Liu X, Rong L, Du R (2021). Ginkgolic acid and anacardic acid are specific covalent inhibitors of SARS-CoV-2 cysteine proteases. Cell Biosci.

[11] Zhu W, Xu M, Chen CZ, Guo H, Shen M, Hu X, Shinn P, Klumpp-Thomas C, Michael SG, Zheng W (2020). Identification of SARS-CoV-2 3CL Protease Inhibitors by a Quantitative High-Throughput Screening. ACS Pharmacol Transl Sci.

[12] Liu P, Liu H, Sun Q, Liang H, Li C, Deng X, Liu Y, Lai L (2020). Potent inhibitors of SARS-CoV-2 3 C-like protease derived from N-substituted isatin compounds. Eur J Med Chem.

[13] Rawson JMO, Duchon A, Nikolaitchik OA, Pathak VK, Hu WS (2021). Development of a Cell-Based Luciferase Complementation Assay for Identification of SARS-CoV-2 3CL^pro^ Inhibitors. Viruses.

[14] Froggatt HM, Heaton BE, Heaton NS (2020). Development of a Fluorescence-Based, High-Throughput SARS-CoV-2 3CL^pro^ Reporter Assay. J Virol.

[15] Riva L, Yuan S, Yin X, Martin-Sancho L, Matsunaga N, Pache L, Burgstaller-Muehlbacher S, De Jesus PD, Teriete P, Hull MV, Chang MW, Chan JF, Cao J, Poon VK, Herbert KM, Cheng K, Nguyen TH, Rubanov A, Pu Y, Nguyen C, Choi A, Rathnasinghe R, Schotsaert M, Miorin L, Dejosez M, Zwaka TP, Sit KY, Martinez-Sobrido L, Liu WC, White KM, Chapman ME, Lendy EK, Glynne RJ, Albrecht R, Ruppin E, Mesecar AD, Johnson JR, Benner C, Sun R, Schultz PG, Su AI, García-Sastre A, Chatterjee AK, Yuen KY, Chanda SK (2020). Discovery of SARS-CoV-2 antiviral drugs through large-scale compound repurposing. Nature.

[16] Ma C, Wang J (2021). Dipyridamole, chloroquine, montelukast sodium, candesartan, oxytetracycline, and atazanavir are not SARS-CoV-2 main protease inhibitors. Proc Natl Acad Sci U S A.

[17] Blanchard J, Elowe N, Huitema C, Fortin P, Cechetto J, Eltis L, Brown E (2004). High-throughput screening identifies inhibitors of the SARS coronavirus main proteinase. Chem Biol.

[18] Korber B, Fischer WM, Gnanakaran S, Yoon H, Theiler J, Abfalterer W, Hengartner N, Giorgi EE, Bhattacharya T, Foley B, Hastie KM, Parker MD, Partridge DG, Evans CM, Freeman TM, de Silva TI, Sheffield, McDanal C, Perez LG, Tang H, Moon-Walker A, Whelan SP, LaBranche CC, Saphire EO, Montefiori DC, COVID-19 Genomics Group (2020). Tracking Changes in SARS-CoV-2 Spike: Evidence that D614G Increases Infectivity of the COVID-19 Virus. Cell.

[19] Wang Z, Schmidt F, Weisblum Y, Muecksch F, Barnes CO, Finkin S, Schaefer-Babajew D, Cipolla M, Gaebler C, Lieberman JA, Oliveira TY, Yang Z, Abernathy ME, Huey-Tubman KE, Hurley A, Turroja M, West KA, Gordon K, Millard KG, Ramos V, Da Silva J, Xu J, Colbert RA, Patel R, Dizon J, Unson-O’Brien C, Shimeliovich I, Gazumyan A, Caskey M, Bjorkman PJ, Casellas R, Hatziioannou T, Bieniasz PD, Nussenzweig MC (2021). mRNA vaccine-elicited antibodies to SARS-CoV-2 and circulating variants. Nature.

[20] Lopez Bernal J, Andrews N, Gower C, Gallagher E, Simmons R, Thelwall S, Stowe J, Tessier E, Groves N, Dabrera G, Myers R, Campbell CNJ, Amirthalingam G, Edmunds M, Zambon M, Brown KE, Hopkins S, Chand M, Ramsay M (2021). Effectiveness of Covid-19 Vaccines against the B.1.617.2 (Delta) Variant. N Engl J Med.

[21] Hay R (2021). An all-out assault on SARS-CoV-2 replication. Biochem J.

[22] Zhang L, Lin D, Sun X, Curth U, Drosten C, Sauerhering L, Becker S, Rox K, Hilgenfeld R (2020). Crystal structure of SARS-CoV-2 main protease provides a basis for design of improved α-ketoamide inhibitors. Science.

[23] Chen Y, Fu Z, Li D, Yue Y, Liu X (2021). Optimizations of a novel fluorescence polarization-based high-throughput screening assay for β-catenin/LEF1 interaction inhibitors. Anal Biochem.

[24] Fu L, Ye F, Feng Y, Yu F, Wang Q, Wu Y, Zhao C, Sun H, Huang B, Niu P, Song H, Shi Y, Li X, Tan W, Qi J, Gao GF (2020). Both Boceprevir and GC376 efficaciously inhibit SARS-CoV-2 by targeting its main protease. Nat Commun.

[25] Vuong W, Khan MB, Fischer C, Arutyunova E, Lamer T, Shields J, Saffran HA, McKay RT, van Belkum MJ, Joyce MA, Young HS, Tyrrell DL, Vederas JC, Lemieux MJ (2020). Feline coronavirus drug inhibits the main protease of SARS-CoV-2 and blocks virus replication. Nat Commun.

[26] Ma C, Hu Y, Townsend J, Lagarias P, Marty M, Kolocouris A, Wang J, Ebselen (2020). Disulfiram, Carmofur, PX-12, Tideglusib, and Shikonin Are Nonspecific Promiscuous SARS-CoV-2 Main Protease Inhibitors. ACS Pharmacol Transl Sci.

[27] Douzi B (2017). Protein-Protein Interactions: Surface Plasmon Resonance. Methods Mol Biol.

[28] Qiao J, Li YS, Zeng R, Liu FL, Luo RH, Huang C, Wang YF, Zhang J, Quan B, Shen C, Mao X, Liu X, Sun W, Yang W, Ni X, Wang K, Xu L, Duan ZL, Zou QC, Zhang HL, Qu W, Long YH, Li MH, Yang RC, Liu X, You J, Zhou Y, Yao R, Li WP, Liu JM, Chen P, Liu Y, Lin GF, Yang X, Zou J, Li L, Hu Y, Lu GW, Li WM, Wei YQ, Zheng YT, Lei J, Yang S (2021). SARS-CoV-2 Mpro inhibitors with antiviral activity in a transgenic mouse model. Science.

[29] Glahn-Martínez B, Benito-Peña E, Salis F, Descalzo A, Orellana G, Moreno-Bondi M (2018). Sensitive Rapid Fluorescence Polarization Immunoassay for Free Mycophenolic Acid Determination in Human Serum and Plasma. Anal Chem.

[30] Levine L, Michener M, Toth M, Holwerda B (1997). Measurement of specific protease activity utilizing fluorescence polarization. Anal Biochem.

[31] Lee C, Degani I, Cheong J, Lee J, Choi H, Cheon J, Lee H (2021). Fluorescence polarization system for rapid COVID-19 diagnosis. Biosens Bioelectron.

[32] Thomas N, Kim S (2011). Potential pharmacological applications of polyphenolic derivatives from marine brown algae. Environ Toxicol Pharmacol.

[33] Rajan D, Mohan K, Zhang S, Ganesan A (2021). Dieckol: a brown algal phlorotannin with biological potential. Biomed Pharmacother.

[34] Karadeniz F, Kang K, Park J, Park S, Kim S (2014). Anti-HIV-1 activity of phlorotannin derivative 8,4‴-dieckol from Korean brown alga *Ecklonia cava*. Biosci Biotechnol Biochem.

[35] Park J, Kim J, Kwon J, Kwon H, Jeong H, Kim Y, Kim D, Lee W, Ryu Y (2013). Dieckol, a SARS-CoV 3CL^pro^ inhibitor, isolated from the edible brown algae *Ecklonia cava*. Bioorg Med Chem.

[36] Ryu Y, Jeong H, Yoon S, Park J, Kim Y, Park S, Rho M, Kim S, Lee W (2011). Influenza Virus Neuraminidase Inhibitory Activity of Phlorotannins from the Edible Brown Alga *Ecklonia cava*. J Agric Food Chem.

[37] Aatif M, Muteeb G, Alsultan A, Alshoaibi A, Khelif B (2021). Dieckol and Its Derivatives as Potential Inhibitors of SARS-CoV-2 Spike Protein (UK Strain: VUI 202012/01): A Computational Study. Mar Drugs.

[38] Adhikari B, Marasini B, Rayamajhee B, Bhattarai B, Lamichhane G, Khadayat K, Adhikari A, Khanal S, Parajuli N (2021). Potential roles of medicinal plants for the treatment of viral diseases focusing on COVID-19: A review. Phytother Res.

[39] Chen Y, Fu Z, Yan G, Lin Y, Liu X (2021). Optimization of expression conditions and determination the proteolytic activity of codon-optimized SARS-CoV-2 main protease in *Escherichia coli*. Chin J Biotech.

[40] Sacco MD, Ma C, Lagarias P, Gao A, Townsend JA, Meng X, Dube P, Zhang X, Hu Y, Kitamura N, Hurst B, Tarbet B, Marty MT, Kolocouris A, Xiang Y, Chen Y, Wang J (2020). Structure and inhibition of the SARS-CoV-2 main protease reveal strategy for developing dual inhibitors against M^pro^ and cathepsin L. Sci Adv.

[41] Lu J, Liu D, Zhou X, Chen A, Jiang Z, Ye X, Liu M, Wang X (2018). Plant natural product plumbagin presents potent inhibitory effect on human cytochrome P450 2J2 enzyme. Phytomedicine.

[42] Du R, Cooper L, Chen Z, Lee H, Rong L, Cui Q (2021). Discovery of chebulagic acid and punicalagin as novel allosteric inhibitors of SARS-CoV-2 3CL^pro^. Antiviral Res.

